# Perceived Experience of the clozapine Treatment Protocol: A Qualitative Study of Reports by Brazilian Patients With Schizophrenia Treated in a University Outpatient Service Specializing in Psychiatry

**DOI:** 10.1002/brb3.71034

**Published:** 2025-11-21

**Authors:** João Batista Alves dos Santos, Francisco Carlos Specian Júnior, Lucas Serra Valladão, Paulo Dalgalarrondo, Clarissa Rosalmeida Dantas, Egberto Ribeiro Turato

**Affiliations:** ^1^ Laboratory of Clinical‐Qualitative Research, Department of Psychiatry, School of Medical Sciences Universidade Estadual De Campinas (UNICAMP) Campinas Brazil; ^2^ Professors At the Department of Psychiatry School of Medical Sciences Universidade Estadual De Campinas (UNICAMP) Campinas Brazil

## Abstract

**Introduction:**

Understanding the meanings people attribute to observed events and lived experiences is to unveil their symbolic universe, thus allowing us to help them better manage their emotions, reactions, and relationships, particularly in clinical settings. Patients with schizophrenia resistant to treatment with first‐ and second‐generation antipsychotics respond well to clozapine; however, this medication presents agranulocytosis as an important side effect. Consequently, the medication protocol requires frequent blood draws to monitor any effects on blood cells. Investigating patients’ perceived emotional experiences of this procedure triggered our study.

**Objective:**

To interpret emotional and/or symbolic meanings attributed by patients with schizophrenia to the clozapine treatment protocol during follow‐up at a university outpatient service specializing in psychiatry.

**Method:**

A clinical‐qualitative study was conducted based on Turato's design. Data were collected by means of in‐depth semi‐directed interviews with open‐ended questions conducted in‐person with participants using clozapine. Sampling followed Fontanella's theoretical information saturation criterion. Data were explored by Faria‐Schutzer's clinical‐qualitative content analysis (CQCA), employing psychodynamic concepts from Medical Psychology.

**Results:**

Analysis of nine interviews resulted in four categories: (1) the frequent blood collections foreseen by the protocol seem to have little impact on patient's symbolization and treatment adherence; (2) “I come here to stay alive”: the frequent blood collections foreseen by the protocol seem to have a good impact on patient's treatment adherence; (3) Reframing a psychiatric illness: the protocol reinforces the embodiment of a medical diagnosis.

**Discussion:**

Faced with a socially and psychologically non‐silent mental disorder, management of schizophrenic symptoms seems to suppress patient symbolization of the blood count collection protocol and the risk of agranulocytosis. Moreover, the protocol appears to promote safety and favor treatment adherence, as well as to provide a greater level of social integration by inserting patients into the health system dynamics.

**Conclusion:**

Despite the repetitive experience of blood collection for rigorous effect control, such a ritual does not seem to bring about symbolizations “more charged” than the chronicity of the disease itself. Therefore, patients benefit from the frequent blood collections, as it secures them from possible social isolation by promoting social interaction. These benefits can help patients to develop new meanings for their life condition.

## Graphical abstract

This qualitative study explores the symbolic meanings attributed by patients with treatment‐resistant schizophrenia (TRS) to the strict clozapine protocol. Drawing on underutilization data, the research investigates how fantasies, fears, and emotional responses may contribute to non‐adherence. Through in‐depth, semi‐structured interviews and clinical‐qualitative content analysis, three categories emerged: (1) routine blood tests have limited symbolic impact; (2) they are also viewed as life‐affirming, supporting adherence; and (3) the protocol reinforces the embodiment of a psychiatric diagnosis. Findings suggest the clozapine protocol symbolic relevance, though for many, this remains only partially internalised.

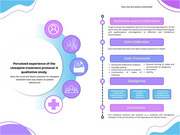

## Significant Outcomes

- Patient adherence to the clozapine protocol is one element among the multifactorial symbolization associated with schizophrenia.
- Exploring emotional meanings in patients with mental disorders is a challenge. In‐person interviews allows to evaluate the unsaid.
- New research on adherence to drug treatment and its association with basic emotional reactions is important for the institutional development of management approaches for health teams.

## Limitations of This Study

- As this research studied the life experiences of people with schizophrenia, a chronic disorder that impairs affective and cognitive functioning, our sample was restricted to those with somewhat preserved psychic and social functions, compatible with the ability to answer an interview about their perceptions of living conditions, and were not currently experiencing a psychotic episode.
- The present study was limited to the analysis of the subjective elements of patients who accepted treatment with clozapine, lacking an examination of the psychodynamic reasons underlying the initial refusal of treatment.
- As this is a qualitative study, the generalization of the results is limited and inherent to this type of research, which holds greater validity when compared with quantitative studies.

## 1 Introduction

Understanding patients’ mental representations about illness and healthcare allows clinical teams for better psychological handling of mentally ill patients, with gains such as greater treatment adherence and prevention. Motivated by the gap identified in the literature regarding qualitative research on life experiences related to clozapine treatment of schizophrenia and the need for recurrent blood collection to assess possible side effects; the absence of works on symbolic emotional meanings attributed to this clinical experience from the perspective of psychodynamic schools; and encouragement to carry out qualitative research in this scope (Pai and Vella 2012), we developed the present research in the fields of Health and Medical Psychology.

Currently, clozapine is the medication of choice for TRS (Haidary and Clozapine 2023), which affects approximately one‐third of patients, with mandatory blood count monitoring throughout the entire treatment period. Agranulocytosis occurs in up to 0.8% of patients and represents a significant medical challenge, despite a reduction in mortality rates (Mijovic and MacCabe 2020).

Drug‐induced agranulocytosis is an idiosyncratic and potentially fatal reaction, characterized by a substantial reduction in neutrophil count, increasing the risk of infections. Clozapine is one of many causative agents, playing an important role in the management of symptoms in TRS, where it is often the only effective option (Siskind et al. 2016). The best way to minimize this potentially fatal side effect is through slow titration and regular blood count monitoring (Keshavan et al. 2022).

Despite being the only medication approved for TRS, clozapine remains widely underutilized. Evidence shows prescription rates ranging from 189.2 per 100,000 individuals in Finland to just 0.6 per 100,000 in Japan (Bachmann et al. 2017), indicating that most countries have utilization rates far below the TRS incidence of 100 per 100,000 (Wimberley et al. 2016). This underutilization of clozapine results in the undertreatment of patients diagnosed with TRS, which may lead to increased morbidity and mortality rates.

This reluctance to prescribe clozapine is often linked to the perceived burden of treatment as well as to a lack of familiarity among clinicians regarding its use (Farooq et al. 2019, Verdoux et al. 2018). Research aimed at understanding these barriers has concluded that patient‐related factors are the primary reason for clozapine underuse. Patient refusal of treatment due to the need for continuous blood monitoring was identified as the main reason in a study involving 144 prescribers in the United Kingdom (Gee et al. 2014).

Based on these findings, it has been established that clozapine treatment is significantly influenced by psychodynamic factors affecting patient adherence (Gee et al. 2014). Since the experience of using a novel medication whose introduction requires weekly blood control, frequent lab trips and follow‐ups, and expectations of good therapeutic results can emotionally and symbolically generate fears, insecurities, and ambivalent feelings that may lead to treatment non‐adherence (Jakobsen et al. 2025, Gangadharan and Tirupati 2023), we were motivated to conduct this study.

## 2 Hypothesis

Recurrent blood collection for rigorous control could lead to an uncomfortable life experience and generate insecurities and ambivalent feelings toward treatment adherence.

## 3 Aim of the study

To interpret the psychological meanings reported in open‐ended interviews by patients with schizophrenia attending at a psychiatric outpatient service about their experiences with the clozapine treatment protocol, highlighting the weekly obligatory blood collection routine.

## 4 Method

As a scientific conception from the human sciences, the clinical‐qualitative method (CQM) seeks to investigate the symbolic emotional meanings attributed to perceived clinical phenomena and to illness and health care experiences, with their languages interpreted by a psychodynamic theoretical framework (Turato 2013). Its three pillars are:

(1) A hippocratic clinical attitude of leaning toward those who suffer, providing people with active listening driven by the desire to care;
(2) A kierkegaardian existentialist attitude of valuing the vital anguish inherent in the human condition;
(3) A freudian psychodynamic attitude of valuing the affective relation between interviewer and interviewee, latent in personal interactions, as well as the use of psychodynamic concepts to interpret the meanings reported.

### 4.1 Ambiance and Acculturation

Initial period to get to know the outpatient service's functioning logic/logistics (team, hierarchy, rooms, schedules, procedures). At the same time, the researcher became acculturated from phenomenological and psychological estrangement to affective and intellectual familiarization.

### 4.2 Personal Preparation

Role‐playing sessions to alternatively rehearse the positions of interviewer and interviewee, carried out among colleagues in the research group.

### 4.3 Data Collection

Use of the SDIOQD—semi‐directed interview with open‐ended questions in‐depth, detailed by Fontanella, Campos, and Turato into (1) triggering question; (2) in‐depth questions; and (3) concluding questions.

### 4.4 Data Treatment

**FIGURE 1:**
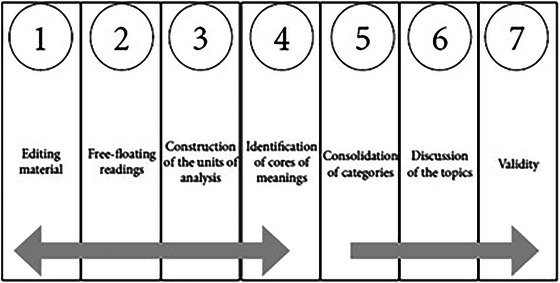
Flowchart of the clinical‐qualitative content analysis In: Faria‐Schützer DB and cols. Seven steps for qualitative treatment in health research: The CQCA. Cien Saude Colet. 2021 Jan; 26(1): 265‐274, p. 269.

Use of the CQCA by Faria‐Schützer et al. (Faria‐Schützer et al. 2021). It is defined by the Seven Steps: (1) Editing the material for analysis; (2) Floating reading; (3) Construction of units of analysis; (4) Construction of codes of meaning; (5) General refining of codes and construction of categories; (6) Discussion; and (7) Validity (see Figure 1).

### 4.5 Theoretical Framework

Medical and Health Psychology, particularly the psychodynamics of relations in clinical settings within the British Balintian framework, illness and falling Supporting Materialill as ways in which people are not healthy in relation to their clinicians, and the desire to become patients in each situation, psychic defense mechanisms against anguish, the ideal of the ego versus the ideal of the ego in patients, family members and clinical team's narcissism, the psychological stages of losses/grief in the illness process, illness as a metaphor in a psychological language of blaming illnesses and patients (Balint 1988, Kübler‐Ross 2017). See Table 1 for the classic ways of understanding and defining non‐health, based on Marinker (Marinker 1975).

**TABLE 1:** Classic ways of understanding and defining non‐health, based in Marinker (Marinker 1975).

Disease ≠ health	Illness ≠ wellness	Sickness ≠ wholeness
Disease, from a biological standpoint, is the objectivity of the pathological process, a deviation from the biomedical norm. Health‐disease manifestations are observed, measured, and correlated, generating medical‐scientific diagnoses and classified in the literature.	Illness, from a psychological understanding, is the personal, intimate feeling of experiencing “poor health,” as subjectively perceived and reported by the individual. It is studied using “qualitative” methods in the search for the psychological meanings attributed to “being unwell” for researchers to understand how it is symbolically constructed by the person.	Sickness, from a sociological perspective, is the organized experience of social coexistence, consisting of a sociocultural manifestation of “non‐health.” Studied using “qualitative” methods in the search for sociological meanings attributed to “feeling sick,” constructed by shared, cultural values, for researchers to understand this collective representation.

^1^The definitions presented in the table highlight different ways of understanding human experience and the process of non‐health, encompassing biological, psychological, and sociological perspectives, based on theoretical concepts with a phenomenological sensitivity.

### 4.6 Research Field Characterization

The study was conducted at an outpatient service of a public university hospital, which provides tertiary care and is a strong research center aligned with the Unified Health System. It is made up of students, residents, supervisors, and nurses who provide daily care to patients from the Metropolitan Area of Campinas, southeastern Brazil. If the researcher was previously part of the team, they underwent a phenomenological/ affective process of defamiliarization.

### 4.7 Participants and Data Collection

The study sample was built intentionally and sequentially by referral from the doctors at the Psychosis and Exits outpatient clinic. The intentional strategy means that we looked for specific personal histories (Balint 1988). Sampling by saturation was used to close the sample and delimit the group of study subjects. Saturation in qualitative research means that no additional data can be extracted from the interviews to construct each category. Theoretical saturation is reached by simultaneous data collection and analysis (Turato 2013, Faria‐Schützer et al. 2021, Fontanella et al. 2011). Participant inclusion criteria consisted of:

(1) Be a patient under follow‐up in the adult psychiatry /psychotic outpatient Clinic from unicamp's HC or under psychiatric hospitalization in the psychiatric ward with a diagnosis of schizophrenia confirmed in the medical record and included in the clozapine protocol;
(2) Have sufficiently experienced the phenomenon under study to be able to report associated emotional meanings;
(3) Have been referred for the interview by a member of the medical team who has been informed about the research and its objectives;
(4) Have autonomously agreed to the interview ethical procedures and principles, corroborated by signing the consent form;
(5) Present adequate physical, emotional, and intellectual conditions, verified by consistency during data collection and by clinical evaluation conducted by the outpatient clinic professors, so as not to jeopardize methodological validity, as is expected when obtaining verbalized information in an open‐ended, clinical‐psychological research interview

## 5 Results

The interviews were conducted by the first author, a male fourth‐year medical student at the time of data collection, with no previous relationship with the study participants. The sample was closed with nine interviewees, when the theoretical categories were saturated from statement excerpts and the cores of meaning were constructed by floating (re)readings. The interviews lasted between 11 and 30 min. See Table 2 for a bio‐socio‐demographic sample characterization.

**TABLE 2:** Bio‐socio‐demographic sample characterization, Campinas SP, Brazil, 2021.

Interviewees	E1	E2	E3	E4	E5	E6	E7	E8	E9
Gender	Male	Male	Female	Male	Female	Male	Mame	Female	Male
Age (in years)	22	22	44	48	44	56	67	31	42
Schooling level	Complete secondary education	Incomplete secondary education	Complete higher education	Complete primary education	Complete higher education	Incomplete primary education	Complete primary education	Complete higher education	Complete primary education
Marital status	Single	Single	Single	Single	Single	Married	Single	Single	Single
Lives with	Parents	Parents and brothers	Parents	Mother and brother	Parents	Wife and sons	Alone	Mom	Parents
Duration of clozapine use	2 months	18 days	1 year and 7 months	22 years	18 days	9 years	7 years	8 years	20 years
Time of diagnosis	6 years	5 years	3 years	28 years	2 years	11 years	40 years	10 years	22 years

^Note: a^ From the beginning until the date of the interview. Interruptions in the treatment were disregarded because they did not prevent the symbolization of the study subject.

For the present paper, we selected three categories: (1) the frequent blood collections foreseen by the protocol seem to have little impact on patient's symbolization and treatment adherence; (2) “I come here to stay alive”: the frequent blood collections foreseen by the protocol seem to have a good impact on patient's treatment adherence; and (3) reframing a psychiatric illness: the protocol reinforces the embodiment of a medical diagnosis

## 6 Discussion

### 6.1 Recurrent Blood Count Collection and its Relativised Symbolization

Faced with the schizophrenia‐associated psychotic conditions, the interviewees show little preoccupation with symbolizing usage of the clozapine protocol. In other words, compliance with the protocol brings about little to no symbolic meaning that structures behaviors as those arising from the clinical manifestations of the disorder. This was made evident by evasions of the triggering question during data collection, which highlighted medication side effects or other management situations and/or clinical manifestations of schizophrenia other than the medication protocol.

E2: Well… the medication has lots of effects, right? It's a heavy, complicated drug. It causes a strong reaction. What I mean is that I don't think I need to be taking these drugs. I think that a tea from a specific plant, a specific tea to help with my head, it would help, and I wouldn't even need to be taking this medication. These meds also make your mouth… salivate. You know when your mouth keeps salivating too much?

We categorized possible perceptions of disease, intersecting their manifestations with patients’ symbolization: silent diseases, unperceived or undiagnosed illness, as in the case, of a middle‐aged patient who does not yet know that he has early‐stage essential hypertension—in this case there is no conflict between the demands of the environment and the patient's own possibilities yet; and non‐silent diseases, which may be psychologically, socially, or psycho‐socially non‐silent. Psychologically non‐silent illnesses are diseases perceived and symbolized by the subject but whose signs may go unnoticed by others. Socially non‐silent diseases, on the other hand, have obvious signs or even stigmas that make them recognizable to other people, even if they do not have exuberant symptoms, such as schizophrenia. In most cases, these may also be perceived and symbolized by the affected individual, making them psychologically non‐silent.

Based on this theorizing, schizophrenia is generally seen as a non‐silent disease, both in psychological and social terms, since its symptoms, both negative and positive, end up modulating stigmatizing behavior in the patient (Dalgalarrondo 2019, Mestdagh and Hansen 2014). By manifesting as a psychologically non‐silent disease, schizophrenia contrasts with the silent, unnoticed side effect of clozapine use—agranulocytosis. It is on the basis of such a clash that the symbolization of schizophrenia overlaps with laboratory controls to prevent agranulocytosis. Additionally, the socially noisy illness ends up causing shame and embarrassing situations for patients during their psychotic episodes and beyond.

E4: Yeah, a lot of onlookers looking on, all standing with their arms crossed looking at me.

E8: [At college], some people didn't want to work with me. It was real hard dealing with it. It was a very difficult situation. I felt like I was alone, you know? That I had to do things all on my own. There was a time when I had to pay someone to help me with classes.

Illness, that is, the intimate/subjective feeling in relation to the disease, is more intense than the imagination and symbolization of other elements, such as the protocol. To conclude the discussion of this category, below are some statements denoting normality in relation to the protocol, to further corroborate the debate:

E6: It doesn't bother me at all. It doesn't get in the way of my day‐to‐day life.

E7: Yeah… I got used to it, you know? It's normal. It's a good thing, you need (the exam) to get the meds. If I refuse to take the blood test, I don't get the medication. And so I go about my daily life.

### 6.2 “Is to Stay Alive, You Know? I Come Here to Stay Alive”: Frequent Blood Count Collection as Motivation for Treatment Adherence

Meaning making can be contradictory and coexistent. Symbolization is polysemic, and findings can oppose each other.

In his work *The Doctor, His Patient and the Illness* (Balint 1988), Balint brings to light the idea that the most frequently used drug in general practice is the doctor himself. He makes it clear that it is not only the bottle of medicine or the blister pack of pills offered for the management of a condition that matters, but, above all, the manner in which they are offered to the patient. In short, “the whole atmosphere in which the drug was given and taken” is a central element in the expected therapeutic outcome, and the doctor–patient relationship is the main promoter of this atmosphere.

The lack of information regarding this frequently used “substance” [the doctor] is described by the author as “disconcerting and disturbing,” especially when contrasted with the wealth of data available about other medications, even those recently introduced to the pharmaceutical market and clinical practice. It is upon this concept, grounded in the medical psychology presented by Balint, that this category is based.

Making blood tests obligatory for receiving treatment with atypical antipsychotics promotes, in this category, an ideal of commitment to health, to the social circle, and to trying to recover functionalities lost in the evolution of the psychotic condition. Self‐worth seems to play a central role in this, as does the subjectivity of duty developed in the physician‐patient relation. Within this protocol, as with any therapy offered by a physician, the establishment of a commitment between physician and patient follows tortuous paths, and its inconsistency can define poor adherence to therapy, which includes the recurrent blood count collection. In contrast, a path established in such a way as to deal with propositions, counterpropositions, offers, acceptances, and presumed rejections in the face of disease manifestations, whether organized or not, is capable of promoting acceptance before a price to be paid by the patient. In this study, the price is the maintenance protocol for clozapine use.

E1: I think it's a treatment thing that I'm doing for myself and to help with my relationship at home, with my parents. I've improved my relationship with my father and mother at home a lot, I'm not so disrespectful toward my father and mother anymore.

E4: It's to stay alive, you know? I come here to stay alive.

E6: It's also good because the doctor told me “You have to take it.” It's a check on platelets. Last month, she said that she'd lowered the number of my platelets. I don't know which one it is, white or red. This lowers immunity. I even got sores in my mouth. I had dental treatment, and the doctor didn't even touch it because of that. I think she said it was low immunity. I must do my duty.

This concludes the central role of establishing a good relationship between physician and patient in establishing the commitment and responsibility vital to protocol adherence.

### 6.3 A Protocol That Reinforces an Embodiment of a Medical Diagnosis

Clozapine use‐associated procedures seem to allow us to measure the immeasurable, to personify that which is non‐personalizable. Psychiatric disorders generally have no presumed physical/organic correspondence, and it is not possible to quantify or note improvement through complementary methodologies, such as blood count tests, blood pressure measurements, or imaging tests. This mismatch between the clinical and biological picture ends up creating the fantasy that undergoing recurrent blood collections for clozapine use qualifies the evolution of schizophrenia. Notably, this fantasy predominates in patients who have not fully understood the purpose of this collection. Other patients, in turn, can verbalize this need for a visualized organic counterpart for schizophrenia.

The statements below were responsible for raising the issue of some patients need to personify the medical diagnosis.

E5: Why am I afraid? I'm in a psychiatrist's office just to take medication. I'm not in a bed with a wound to be healed. Understand? I'm only taking [the medication] for the psychological side, that's what's wrong. I'm not here because my arm broke. Or because I have a wound on my arm, okay? I'm not seeing the day‐to‐day dressing as the guy who has a tracheostomy. He… every day you see that he's [tracheostomized], not now, now they've covered it up. He's already in the process of healing, the hole is already mending. It's different, it's different. Now for me, it's just psychological…

Entering the clozapine protocol materializes mental illness. The numbers, the cells affected or not, the texture of the paper on which the test results are presented, the doctor's words denoting the normality of the test for a psychopathology in which normality has never been descriptive enough are elements of intense symbolization. This is because they turn the disease into a measurable phenomenon, giving consistency to an element of the mind. In other words, the materiality of carrying out a rigid protocol helps in the mental construction of the symbolizations resulting from schizophrenia.

The protocol for clozapine use, by promoting frequent contact with the nursing team and patients’ travel to the hospital, ends up having a secondary benefit in the social, affective, and even cognitive rehabilitation of patients with schizophrenia, by allowing systematic desensitization. Below are some interview excerpts that allowed us to reach this conclusion:

E4: I like it because I don't talk to anyone. I'm not like my neighbor who chats with the neighbour next door or with the other one down the street. I live alone at home.

E9: It's good to get out of the house a bit too, I come here to talk. It's really nice. I stay at home all day. I get up, get ready, do my things, pick up my cell phone and check the time. When it gets close to the time, I tell my mother that we must go to Unicamp. So we leave and come here, I go to the appointment, and everything, everything's fine.

The interviews above show that breaking away from the social isolation often faced by patients with schizophrenia is a key factor in restructuring their social, affective, and cognitive lives. This break is induced by the clozapine protocol, which creates the need for travel and social interaction, promoting systematic desensitization to patients’ affective, social, and even cognitive dullness, as well as raising the need for organization and preparation for the appointment, with possible cognitive benefits.

## 7 Conclusions

Patients attribute significant therapeutic value to clozapine due to the protocol‐based monitoring it entails, including frequent full blood counts. The severity and chronic nature of schizophrenia lead to a more structured mental representation of daily life, stemming from the fear of compromising one's mental health. This detailed protocol for the detection of potential agranulocytosis underscores the importance of proper adherence to the medication. Schizophrenia is thus reframed as a condition requiring continuous care.

Our findings may help elucidate the psychodynamic factors involved in the acceptance of clozapine treatment, which still encounters resistance from some healthcare professionals and patients. In terms of practical applications, the results can be utilised in the psychological management by doctors and nurses in clinical settings with patients undergoing treatment, by providing theoretical and psychodynamic support for the subjectivity involved in this process.

Regarding theoretical implications, our findings may inform the training of medical residents and continuing education programmes for experienced professionals, as well as institutional policies aimed at the treatment of schizophrenia. Future qualitative research could include the analysis of patients who refused clozapine treatment, to explore potential fears or fantasies underlying their refusal, which continue to contribute to the underuse of what is considered the medication of choice for TRS. When combined with the present work, which focuses on patients who accepted the treatment, such research could further clarify the subjective elements involved and contribute more comprehensively to the optimisation of clinical management.

## Author Contributions

**João Batista Alves dos Santos**: conceptualization, data curation, formal analysis, funding acquisition, investigation, validation, visualization, writing – original draft preparation, and writing – review & editing. **Francisco Specian Júnior**: conceptualization, methodology, and writing – original draft preparation. **Lucas Serra Valladão**: conceptualization, formal analysis, and validation. **Paulo Dalgalarrondo**: project administration, supervision, and validation. **Clarissa Rosalmeida Dantas**: conceptualization, project administration, supervision, and validation. **Egberto Ribeiro Turato**: conceptualization, data curation, formal analysis, funding acquisition, methodology, project administration, resources, supervision, validation, and visualization.

## Funding

Grant number 2021/08084‐5, São Paulo Research Foundation (FAPESP), Brazil.

## Disclosure

The abstract from this article was presented at the European Congress of Psychiatry, Paris, France, 2023.

## Ethics Statement

Ethical approval granted by the Ethics Committee of the Universidade Estadual de Campinas (UNICAMP), Brazil, Protocol number 5,132,832, Campinas SP, Brazil, November 29th, 2021.

## Conflicts of Interest

The authors declare no conflicts of interest.

## Supporting information




**Supplementary Material**: brb371034‐sup‐0001‐SuppMat.docx

## Data Availability

The data set that support our findings are available upon request to the corresponding author. The data are not publicly available due to privacy or ethical restrictions.
