# Supplementary material for: Perceived Experience of the clozapine Treatment Protocol: A Qualitative Study of Reports by Brazilian Patients With Schizophrenia Treated in a University Outpatient Service Specializing in Psychiatry
Source: Brain Behav. 2025 Nov 21;15(11):e71034. doi: 10.1002/brb3.71034 (PMC12638437; doi:10.1002/brb3.71034)
Supplement: Supplementary file 1 — Supplementary Material: brb371034‐sup‐0001‐SuppMat.docx [file BRB3-15-e71034-s001.docx]

**Contributor Role Taxonomy (CRediT):**

**João Batista Alves dos Santos** (ORCID: 0000-0001-5990-5152) – email: [jbatistaalves16@gmail.com](mailto:jbatistaalves16@gmail.com)

- Conceptualization
- Data Curation
- Formal Analysis
- Funding Acquisition
- Investigation
- Validation
- Visualization
- Writing – Original Draft Preparation
- Writing – Review & Editing

**Francisco Specian Júnior** (ORCID: 0000-0002-4220-2642) – email: [franciscotga@hotmail.com](mailto:franciscotga@hotmail.com)

- Conceptualization
- Methodology
- Writing – Original Draft Preparation

**Lucas Serra Valladão** (ORCID: 0000-0002-8448-7236) - email: [lucassvalladao@gmail.com](mailto:lucassvalladao@gmail.com)

- Conceptualization
- Formal Analysis
- Validation

**Paulo Dalgalarrondo** (ORCID: 0000-0001-9870-6391) – email: [pdalga@unicamp.br](mailto:pdalga@unicamp.br)

- Project Administration
- Supervision
- Validation

**Clarissa Rosalmeida Dantas** (ORCID: 0000-0001-8480-2585) – email:claris63@unicamp.br

- Conceptualization
- Project Administration
- Supervision
- Validation

**Egberto Ribeiro Turato** (ORCID: 0000-0002-7857-1482) – email: [egberto@unicamp.br](mailto:egberto@unicamp.br)

- Conceptualization
- Data Curation
- Formal Analysis
- Funding Acquisition
- Methodology
- Project Administration
- Resources
- Supervision
- Validation
- Visualization
